# Income insecurity and social protection: Examining the impact of the COVID-19 pandemic across income groups

**DOI:** 10.1371/journal.pone.0310680

**Published:** 2024-09-20

**Authors:** Na Chen

**Affiliations:** School of Government, Beijing Normal University, Beijing, China; Kodolanyi Janos University of Applied Sciences: Kodolanyi Janos Egyetem, HUNGARY

## Abstract

The COVID-19 pandemic has wrought unprecedented disruption on global economies, leading to widespread income insecurity among individuals and households. This study investigates the impact of the pandemic on income insecurity across different income groups and assesses the role of social protection measures in mitigating these effects. Using cross-country data, we analyze the prevalence of income reduction and the effectiveness of social assistance programs in high-income, upper middle-income, lower middle-income, and low-income countries. Our findings reveal significant variations in income insecurity and social protection responses across these groups. the pandemic had a significant impact on household incomes globally, with lower-middle-income countries experiencing the most significant income reductions. The average per capita transfer amounts show a general decrease over time, which could be due to the economic strain on governments and the need for more sustainable social protection programs. The correlation between transfer amounts and the proportion of households with reduced income indicates that countries with higher income reduction rates tended to have lower average per capita transfer amounts, suggesting a potential lack of adequate support for those in need. The study highlights the importance of robust social safety nets in cushioning the economic blow of the pandemic, particularly for vulnerable populations in lower-income countries.

## 1. Introduction

The COVID-19 pandemic, declared by the World Health Organization (WHO) in March 2020, has emerged as the most significant global health crisis in recent history, with far-reaching economic and social consequences. The rapid spread of the virus has prompted governments worldwide to implement strict measures, including lockdowns and social distancing, to contain the spread of the disease. These measures, while necessary from a public health perspective, have had a profound impact on economic activity, leading to job losses, reduced working hours, and business closures, which in turn have increased income insecurity for millions of individuals and households. The COVID-19 pandemic has had a profound impact on household incomes globally, with many governments facing significant fiscal challenges in maintaining income support programs. This paper explores the role of social protection theories in understanding the government response and the implications for household vulnerability and financial stability.

### 1.1. Background to the COVID-19 pandemic

The COVID-19 pandemic has affected over 200 countries and territories, causing a global economic downturn that has been compared to the Great Depression of the 1930s. The International Monetary Fund (IMF) and the World Bank have both reported a contraction in global GDP unprecedented in peacetime. The pandemic has exposed the vulnerabilities of global supply chains, the dependence on international trade, and the risks inherent in the gig economy and informal labor markets.

The COVID-19 pandemic has emerged as one of the most significant global crises of the 21st century, affecting the lives of billions and reshaping the social, economic, and political landscapes of countries worldwide. One of the most pressing issues brought to the fore by the pandemic is income insecurity, which has seen a sharp rise as economies have contracted, jobs have been lost, and livelihoods have been disrupted. In response, social protection systems have become a critical component in mitigating the immediate and long-term impacts of this crisis on individuals and communities.

This study aims to examine the impact of the COVID-19 pandemic on income insecurity across different income groups and to assess the effectiveness of social protection measures in providing a safety net for those affected. The introduction of the novel coronavirus and the subsequent policy responses have highlighted both the strengths and the fragilities of existing social protection frameworks. Understanding how these systems have fared during the pandemic is essential for informing future policy design and ensuring that the most vulnerable members of society are adequately protected during times of crisis.

### 1.2. The concept of income insecurity

Income insecurity refers to the uncertain ability of individuals or households to maintain a stable and adequate income to meet their basic needs and sustain their standard of living. It is often associated with a lack of financial resilience and can lead to poverty, food insecurity, and other socio-economic challenges. The COVID-19 pandemic has exacerbated income insecurity due to the sudden loss of income for many workers, particularly those in sectors directly affected by lockdown measures, such as tourism, hospitality, and retail.

Income insecurity is a multifaceted issue that encompasses not only the absence of income but also the unpredictability and inadequacy of income to meet basic needs. The pandemic has exacerbated these challenges, with workers in informal sectors, low-skilled jobs, and those in lower-income brackets being particularly hard hit. The crisis has underscored the importance of social protection as a tool for reducing poverty, inequality, and vulnerability.

### 1.3. The role of social protection in times of crisis

Social protection programs are designed to provide support to individuals and families facing economic hardship, poverty, and social exclusion. These programs include cash transfers, unemployment benefits, pensions, and other forms of assistance aimed at reducing poverty and inequality. During the COVID-19 crisis, social protection has become even more critical, serving as a lifeline for many households affected by the economic downturn.

### 1.4. Research objectives and questions

This paper aims to examine the impact of the COVID-19 pandemic on income insecurity across different income groups and assess the effectiveness of social protection measures in mitigating these effects. The research questions addressed in this study include:

- How has the COVID-19 pandemic affected income insecurity across different income groups?
- What role have social protection programs played in reducing income insecurity during the crisis?
- Are there differences in the social protection response between high-income, upper middle-income, lower middle-income, and low-income countries?
- What lessons can be learned from the experiences of different countries in terms of enhancing social protection systems to better respond to future crises?

By answering these questions, this study seeks to contribute to the understanding of income insecurity during the COVID-19 pandemic and provide insights into how social protection systems can be strengthened to promote income security and resilience in the face of future challenges.

## 2. Literature review

This section of the paper reviews the existing literature on income insecurity and social protection, providing a foundation for the study’s theoretical frameworks and highlighting the gaps that this research aims to fill.

### 2.1. Previous studies on income insecurity

Income insecurity has been a subject of interest in the social sciences for decades, with research often focusing on the causes and consequences of economic vulnerability. Previous studies have explored the links between income insecurity and various socio-economic outcomes, including health [1, 2], mental health [3, 4], and interpersonal relationships [5, 6]. The literature has also examined the role of economic policies and labor market structures in shaping income security.

The COVID-19 pandemic has had a profound impact on global economies, leading to widespread income insecurity and job losses. This literature review examines the scholarly research on the impact of the pandemic on income groups and the role of social protection programs in mitigating these effects. It identifies gaps in current knowledge and highlights the insufficiencies of previous studies in fully capturing the complexity of the crisis [7–9].

The COVID-19 pandemic has exposed and exacerbated income inequalities across the world. Lockdowns and restrictions on economic activity have led to job losses and reduced income for many, with the burden falling disproportionately on certain income groups [10, 11]. Social protection programs have been crucial in providing support to affected individuals and households, but their effectiveness varies widely between countries and income groups [12–14].

Previous studies have largely focused on the immediate impact of the pandemic on employment and income, with some examining the role of social protection programs. However, these studies often suffer from methodological limitations and do not fully capture the long-term consequences of the crisis or the differential impact on various income groups.

Research has shown that low-income and lower-middle-income groups have been hardest hit by the pandemic, experiencing higher rates of job loss and income reduction. These groups often lack access to social protection programs and savings to buffer against economic shocks [15–18]. In contrast, high-income groups have been better able to maintain their income levels, often through remote work opportunities.

Social protection programs have played a critical role in providing financial support to affected individuals and households. Studies have found that countries with robust social safety nets have been better able to mitigate the impact of the pandemic on income insecurity [19–21]. However, there is significant variation in the effectiveness of these programs between countries, with some failing to provide adequate support to those most in need.

Many previous studies have failed to adequately consider the differential impact of the pandemic on various income groups. Additionally, there is a lack of research examining the long-term consequences of the crisis on income insecurity and the effectiveness of social protection programs in promoting recovery [22–25].

This literature review highlights the need for more comprehensive and nuanced research on the impact of the COVID-19 pandemic on income groups and the role of social protection programs. Future studies should examine the long-term consequences of the crisis, the differential impact on various income groups, and the effectiveness of social protection programs in promoting recovery and reducing income inequalities.

The COVID-19 pandemic has had a profound impact on income insecurity, with certain income groups being more severely affected than others. Social protection programs have played a crucial role in mitigating the impact of the crisis [26, 27], but there is significant room for improvement [28–31]. Further research is needed to fully understand the complex dynamics of the pandemic and to inform evidence-based policy responses to promote more equitable and resilient societies.

### 2.2. Theoretical frameworks of social protection

The theoretical framework for this study is based on the social investment perspective and the social insurance perspective. The social investment perspective emphasizes the long-term benefits of investing in individuals, while the social insurance perspective focuses on the role of collective risk management through social insurance programs.

**Fig 1:**
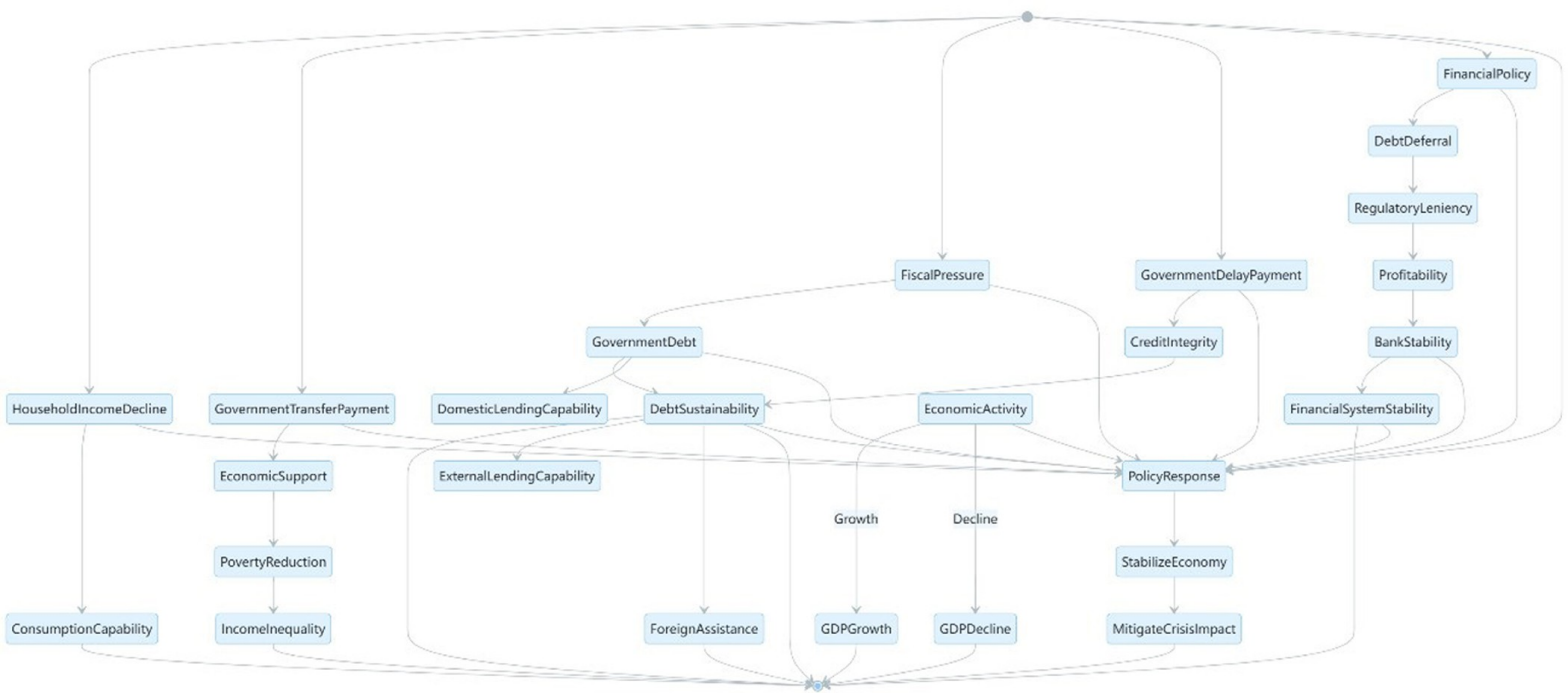
Theoretical frameworks of social protection.

Social protection theories provide a framework for understanding the principles and objectives behind social policy interventions. Key theories include the social insurance approach, which emphasizes risk pooling and protection against life-cycle events; the social assistance approach, which targets support to the most vulnerable; and the social investment approach, which focuses on enhancing human capital and productivity. These frameworks inform the design and implementation of social protection programs in Fig 1.

### 2.3. Global responses to economic crises

The global response to economic crises, including the 2008 financial crisis and the current COVID-19 pandemic, has involved a range of social protection measures. Studies have examined the effectiveness of these responses in terms of mitigating the impact of crises on poverty and inequality. The literature also explores the challenges faced by countries in implementing expansive social protection programs, particularly in the context of limited resources and fiscal constraints.

### 2.4. Gaps in the literature and research contributions

Despite the extensive literature on income insecurity and social protection, there are gaps that this study aims to address. Firstly, there is a need for more research examining the differential impact of economic crises on income insecurity across different income groups. Secondly, while the role of social protection in crises is recognized, there is a lack of comparative studies on the effectiveness of social protection responses across high-income, middle-income, and low-income countries. This study aims to contribute to filling these gaps by providing a cross-country analysis of income insecurity and social protection during the COVID-19 pandemic.

## 3. Methodology

This section outlines the research design, data sources, and analytical methods used to investigate the impact of the COVID-19 pandemic on income insecurity across different income groups and the role of social protection in mitigating these effects.

### 3.1. Data sources and collection

The primary data for this study is drawn from international organizations such as the World Bank, the International Monetary Fund (IMF), and the United Nations (UN) agencies, which have been monitoring and reporting on the socio-economic impacts of the COVID-19 pandemic. The research design includes a comparative analysis of income support programs in countries that were hit by multiple waves of the pandemic, lacked strong automatic stabilizers, and were unable to mobilize external fiscal resources. The data is collected from the World Bank ASPIRE (Atlas of Social Protection Indicators of Resilience and Equity), and the World Bank COVID-19 Household Monitoring Dashboard. Additionally, national statistical agencies and government reports are consulted for country-specific data on income, social protection programs, and policy responses to the crisis. Data on household income, employment status, and social assistance benefits are particularly relevant for this analysis.

1) Assumption: The strength of social safety nets (measured by per capita transfer payments) is negatively correlated with the proportion of households experiencing income reduction.

Verification Question: Can a stronger social safety net effectively reduce household income losses during crises?

2) Assumption: Households in high-income countries experience a lower proportion of income reduction during the COVID-19 crisis.

Verification Question: Do households in high-income countries suffer less impact due to stronger social safety nets?

3) Assumption: The proportion of income reduction for households in low-income countries is negatively correlated with the strength of the country’s social safety net.

Verification Question: Can social safety nets in low-income countries effectively mitigate household income losses?

### 3.2. Selection of countries and income groups

The study selects a sample of countries representing the four income groups as defined by the World Bank: high income, upper middle income, lower middle income, and low income. The aim is to include countries from various regions to ensure a broad and comparative perspective.

The countries included in this study were selected based on data availability, representativeness, and the diversity of social protection systems. The sample includes a mix of high-income, upper middle-income, lower middle-income, and low-income countries, representing various regions and levels of development. The income groups were defined using the World Bank’s income classification system, which divides countries into four categories: high income, upper middle income, lower middle income, and low income. This classification is based on the country’s gross national income (GNI) per capita.

### 3.3. Variables and definitions

- Income Insecurity: Measured as the percentage of households experiencing a reduction in income due to the pandemic.
- Social Protection Response: Captured by the average per capita transfer from social protection programs and the percentage of households receiving such assistance.
- Economic Indicators: Such as GDP per capita, unemployment rates, and the poverty rate, to provide context for the analysis.

ISO3 Code: The ISO3 code is a three-letter code assigned to countries by the International Organization for Standardization (ISO). It is used as a standard identifier for countries in international statistics and data analysis. Because these countries represent different income levels and regions, which helps to assess the impact of the pandemic under different socio-economic backgrounds. For example, high-income countries may have stronger social protection systems, while low-income countries may face greater economic pressures.

Income Group: The income group classification is based on the World Bank’s income classification system, which divides countries into four categories: high income, upper middle income, lower middle income, and low income. The classification is based on the country’s gross national income (GNI) per capita. Analyzing internal inequalities can be done by comparing different income groups or regions.

Share of Households that Reduced Income: This variable measures the percentage of households in each country that reported a reduction in income due to the COVID-19 pandemic. The data is likely collected through household surveys or other primary data sources.

Average Per Capita Transfer: This variable represents the average amount of money per person that households received from social protection and labor programs during the pandemic. The data is likely collected through administrative records or surveys, and the transfer amounts are aggregated to the per capita level.

Average per capita transfer payments can reflect the efficiency and coverage of social protection systems, while the percentage of households that have reduced income directly reflects economic pressure. These indicators together provide a comprehensive assessment of the response capabilities of social protection and labor departments during the pandemic.

### 3.4. Analytical framework and statistical methods

The analytical framework for this study is built upon the economic and social theories of income insecurity and social protection. It incorporates concepts such as the economic vulnerability of households, the role of social safety nets in times of crisis, and the impact of policy responses on income distribution. The framework guides the selection of variables, the development of hypotheses, and the interpretation of results.

In terms of statistical methods, the analysis primarily involves descriptive statistics to summarize the data, such as calculating averages, medians, and standard deviations for the average per capita transfer. Regression analysis is used to examine the relationships between income insecurity and social protection, controlling for factors such as income group, economic indicators, and the year of the indicator. This allows for the identification of trends and correlations that may not be apparent in the raw data.

Additionally, comparative analysis is employed to identify patterns and differences across income groups. This involves the use of t-tests or ANOVA to compare means and proportions, as well as chi-square tests to assess the association between categorical variables.

The research also employs a mixed-methods approach, combining quantitative analysis of large datasets with qualitative analysis of policy documents and case studies. This provides a comprehensive understanding of the dynamics of income insecurity and social protection during the pandemic.

By employing these methods, the study aims to provide evidence-based insights into the effectiveness of social protection measures in addressing income insecurity across different income groups and to inform policy recommendations for future crisis response.

The analytical framework is grounded in the economic and social theories of income insecurity and social protection. Statistical methods employed include descriptive statistics to summarize the data, regression analysis to examine the relationships between income insecurity and social protection, and comparative analysis to identify patterns and differences across income groups. Multivariate analysis may also be used to control for confounding factors that could influence the outcomes.

The study uses a mixed-methods approach, combining quantitative analysis of large datasets with qualitative analysis of policy documents and case studies to provide a comprehensive understanding of the dynamics of income insecurity and social protection during the pandemic. Endogeneity issues can be mitigated by controlling variables or using instrumental variables. GDP growth rate of a country has be controlled as it may simultaneously affect household income and transfer payments.

**Fig 2:**
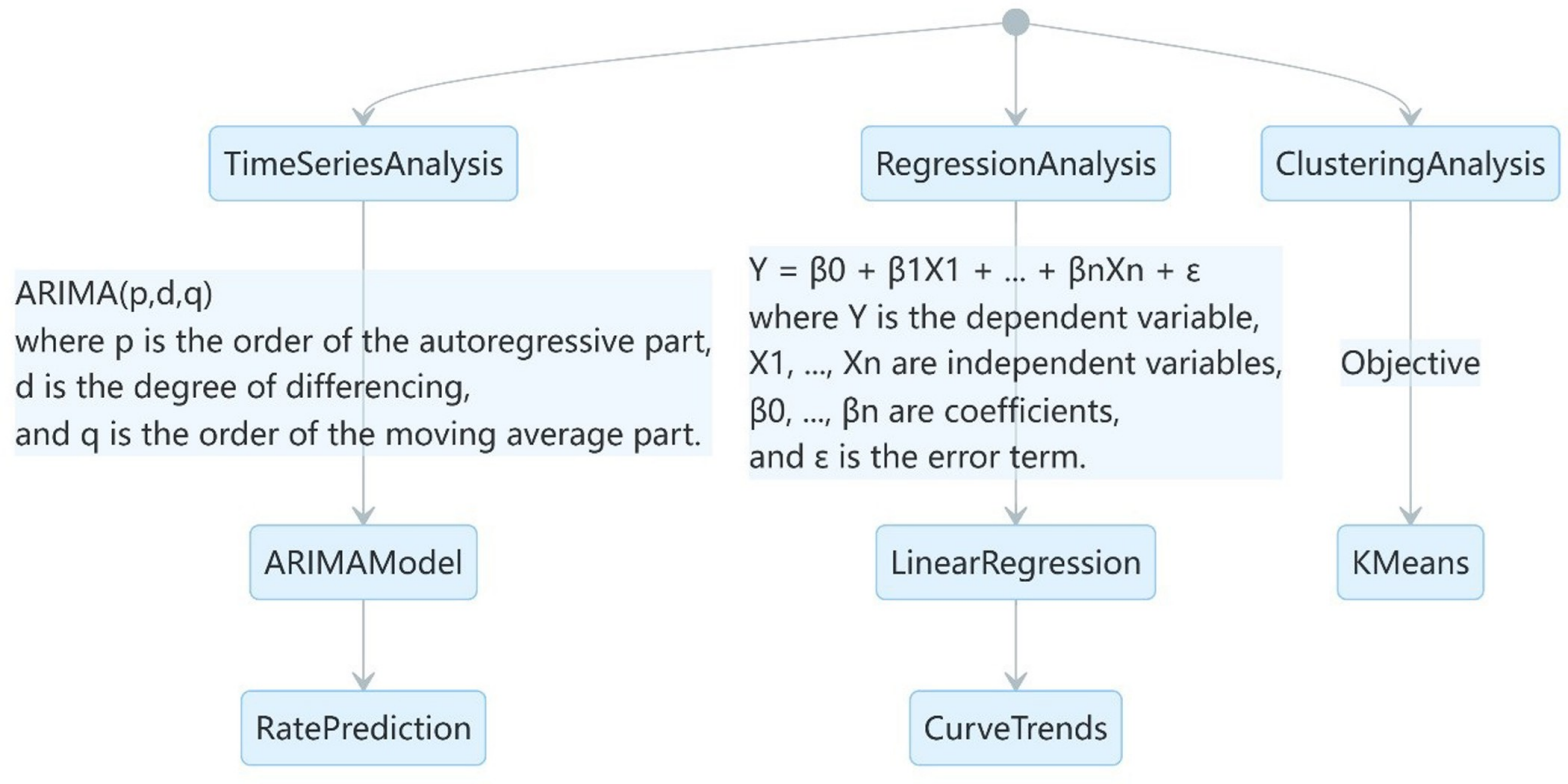
Analytical framework and statistical methods.

By employing these methods, the study aims to provide evidence-based insights into the effectiveness of social protection measures in addressing income insecurity across different income groups and to inform policy recommendations for future crisis response in Fig 2.

## 4. Global income insecurity in the wake of the COVID-19 pandemic

This chapter examines the global landscape of income insecurity following the outbreak of the COVID-19 pandemic, considering the pre-pandemic trends, the impact of lockdowns and economic slowdowns, and the variations in income insecurity across different income groups.

### 4.1. Pre-pandemic income insecurity trends

Before the pandemic, income insecurity was already a concern for many countries, with rising inequality and the proliferation of informal labor markets contributing to economic vulnerability. This section reviews the trends in income insecurity, including the prevalence of poverty, the growth of the gig economy, and the challenges faced by developing nations in providing social safety nets.

### 4.2. The impact of lockdowns and economic slowdowns

The COVID-19 pandemic led to widespread lockdowns and restrictions on economic activity, which had a immediate and severe impact on global economies. This section analyzes how these measures affected employment, productivity, and income levels, leading to a surge in income insecurity. The focus is on the short-term consequences of the pandemic on household incomes and the subsequent increase in demand for social protection.

### 4.3. Income insecurity across income groups

Income insecurity has not affected all countries equally, with variations observed across different income groups. This section compares the experiences of high-income, upper middle-income, lower middle-income, and low-income countries. It explores the factors that have exacerbated or mitigated income insecurity in each group, such as the capacity for fiscal stimulus, the robustness of social protection systems, and the reliance on informal labor markets. The analysis highlights the disparities in income insecurity and the role of social protection in addressing these disparities during the pandemic.

By examining these aspects, the chapter aims to provide a nuanced understanding of the impact of the COVID-19 pandemic on income insecurity across different income groups and to identify the factors that have influenced the outcomes for income insecurity.

## 5. Social protection responses to the pandemic

This section delves into the global social protection responses to the COVID-19 pandemic, examining the range of measures implemented, highlighting effective strategies through case studies, and discussing the variations in social protection approaches across different income groups.

### 5.1. Overview of global social protection measures

The COVID-19 pandemic has led to the implementation of various social protection measures worldwide. This section provides a comprehensive overview of these measures, including cash transfers, unemployment benefits, food aid, and health insurance coverage. The overview also discusses the scalability and adaptability of these programs in the face of a rapidly evolving crisis.

### 5.2. Case studies of effective social protection strategies

This subsection presents in-depth case studies of countries that have implemented particularly effective social protection strategies during the pandemic. It examines the components of these strategies, such as the speed of implementation, the targeting of beneficiaries, and the integration of social protection with health and economic policies. The case studies serve to illustrate best practices and lessons learned for other countries.

- **High-income countries** like Chile (CHL) and Mauritius (MUS) show a significant average per capita transfer, which might indicate a robust social safety net. Poland (POL) has an exceptionally high average per capita transfer of over 10 units, suggesting a strong government response.
- **Upper middle-income countries** vary widely in their responses. For instance, Argentina (ARG) and Colombia (COL) have higher average per capita transfers compared to others like Dominican Republic (DOM) and Paraguay (PRY).
- **Lower middle-income countries** generally show lower average per capita transfers, with Ghana (GHA) and Malawi (MWI) having very low values, which might imply limited capacity for social safety net support.
- **Low-income countries** like Ethiopia (ETH) and Malawi (MWI) have a high percentage of households with reduced income but relatively low average per capita transfers, indicating a significant impact on the population with less government support.
- **Outliers** include Uzbekistan (UZB) with a relatively high average per capita transfer for a lower middle-income country, and Georgia (GEO) with a low percentage of households with reduced income compared to others in the same income group.

### 5.3. Variations in social protection by income group

Social protection responses have varied significantly across different income groups. High-income countries, with more resources and established social safety nets, have been able to provide substantial support to their citizens. Upper middle-income countries have faced challenges in balancing economic constraints with the need for robust social protection. Lower middle-income and low-income countries have had to be more innovative, often with limited resources and less developed social protection systems. This subsection analyzes these variations, exploring the factors that have influenced the effectiveness of social protection responses in each income group.

By examining these aspects, the chapter aims to provide a nuanced understanding of how social protection has been used globally to combat income insecurity during the COVID-19 pandemic and to identify strategies that can be replicated or adapted in future crisis response efforts.

## 6. Household income reduction and social assistance

This chapter focuses on the direct impact of the COVID-19 pandemic on household incomes and the role of social assistance programs in mitigating the resulting economic hardship.

### 6.1. The prevalence of income reduction

The first section of this chapter presents a detailed analysis of the prevalence of income reduction among households across the selected countries. It examines the extent to which households in different income groups have experienced a decrease in income as a result of the pandemic. The analysis considers various factors that may have influenced the scale of income reduction, such as the severity of lockdown measures, the structure of the economy, and the reliance on affected sectors.

1. National Income Classification covers countries with different income levels ranging from high to low, which helps us understand the situation of social security and income losses under different economic conditions.
  - High-income countries include CHL (Chile), MUS (Mauritius), POL (Poland), etc., while low-income countries include ETH (Ethiopia), MWI (Malawi), etc.
2. Proportion of Households Experiencing Income Reduction:
  - Most countries reported relatively high proportions of households experiencing income reduction, reflecting the widespread impact of the COVID-19 pandemic on the global economy.
  - Both low-income countries (such as MWI, ETH) and high-income countries (such as CHL, CRI) faced high income reduction proportions, highlighting the universal impact of the pandemic on the global economy.
  - The income reduction proportions in middle-income countries (including upper-middle and lower-middle income countries) are also generally high, indicating that the impact of the pandemic spans multiple income levels.
3. Average Per Capita Transfers for Social Security and Labor:
  - High-income countries (such as MUS, POL) have higher average per capita transfer amounts, which may reflect their stronger social security systems and more extensive labor protection policies.
  - Low-income countries generally have lower average per capita transfer amounts, reflecting their limited economic resources and weaker social security systems.
  - Some middle-income countries (such as ARG, CRI) also reported relatively high average per capita transfer amounts, indicating that they took active social security measures during the pandemic.
4. Year Differences:
  - The years for the average per capita transfer indicators vary, reflecting differences in data availability and reporting times across countries.
  - Despite the inconsistent years, considering that the COVID-19 pandemic broke out in early 2020, these years’ data can still provide us with useful information about the social security and labor protection situations in various countries.
5. Conclusion:
  - The impact of the COVID-19 pandemic on the global economy is universal and profound, with challenges in family income reduction regardless of the country’s income level.
  - High-income countries typically have stronger social security systems and more extensive labor protection policies, which help mitigate the impact of the pandemic on families and individuals.
  - Middle-income and low-income countries have also taken some social security measures during the pandemic, but these measures may be limited by economic resources and administrative capabilities.
  - Continued attention needs to be paid to the social security and labor protection situations in different countries in the future, and international cooperation should be strengthened to jointly address global challenges such as pandemics.

### 6.2. The role of social assistance in mitigating income loss

**Fig 3:**
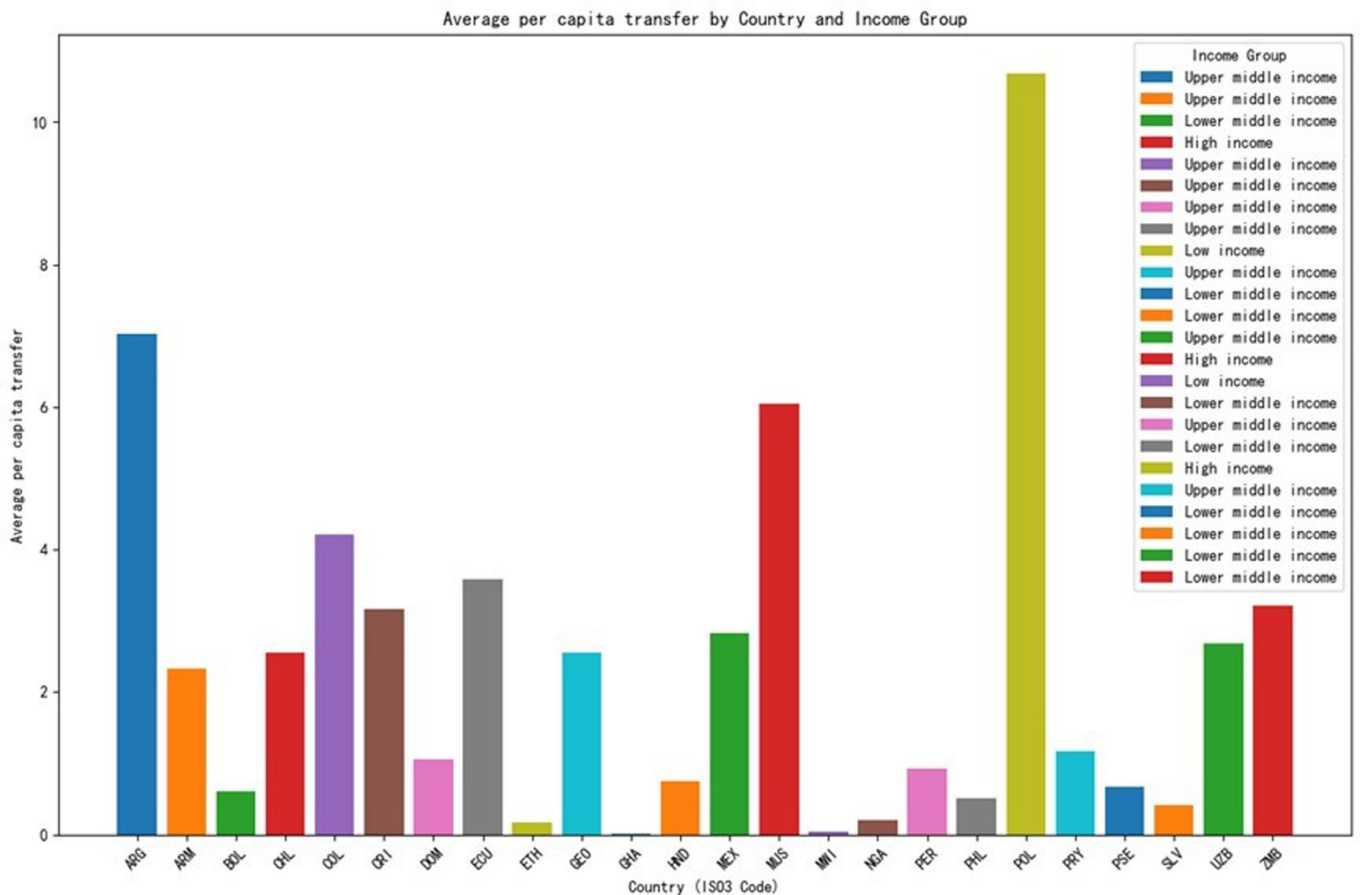
Average per capita transfer amounts in different countries.

Here, the focus shifts to the role of social assistance programs in providing a safety net for households affected by income loss. The chapter explores how different types of social assistance, such as cash transfers, unemployment benefits, and food aid, have helped to offset the economic impact of the pandemic. The effectiveness of these programs in reducing poverty and inequality during the crisis is evaluated, and the chapter discusses the importance of a comprehensive and integrated approach to social protection in Fig 3.

As shown in the chart above, we can observe the average per capita transfer amounts in different countries. Each country’s bar represents its average per capita transfer amount, and the different colors indicate the different income groups. From the chart, it is evident that the average per capita transfer amounts in high-income countries are generally higher than those in middle- and low-income countries. This trend demonstrates a clear correlation between income levels and the level of per capita transfer amounts among countries.

### 6.3. Country-level analysis of income reduction and social assistance

The final section of this chapter conducts a comparative analysis of country-level data to assess the relationship between income reduction and social assistance. It examines how the prevalence of income reduction correlates with the scale and reach of social assistance programs. The analysis also considers the policy responses of individual countries and how these have influenced the outcomes for households. Case studies may be used to illustrate the diversity of approaches and their outcomes.

We can draw several conclusions regarding social safety nets and income losses during the COVID-19 crisis across different country income groups:

1. High-income Countries: High-income countries like Chile (CHL) and Mauritius (MUS) have a relatively high average per capita transfer, indicating a strong social safety net response. Poland (POL) stands out with the highest average per capita transfer of over 10 units, suggesting a substantial government effort to support its citizens.
2. Upper Middle-income Countries: There is a wide variation in the response of upper middle-income countries. Argentina (ARG) and Colombia (COL) show higher average per capita transfers compared to others in this group, such as Dominican Republic (DOM) and Paraguay (PRY), indicating different capacities and strategies to address the crisis.
3. Lower Middle-income Countries: Lower middle-income countries, with a few exceptions, generally have lower average per capita transfers. Ghana (GHA) and Malawi (MWI) have very low average per capita transfers, which may reflect limited resources for social safety net programs.
4. Low-income Countries: Ethiopia (ETH) and Malawi (MWI) have a high percentage of households with reduced income and low average per capita transfers, suggesting that these countries faced significant economic challenges with limited resources to provide substantial support.
5. Outliers: Some countries show unique patterns. Uzbekistan (UZB) has a relatively high average per capita transfer for a lower middle-income country, which might indicate a prioritization of social safety net programs. Georgia (GEO) has a low percentage of households with reduced income compared to other upper middle-income countries, potentially reflecting resilience in its economy or effective government policies.
6. Economic Impact: The percentage of households that reduced their income varies widely across countries, with some countries like Honduras (HND), Nigeria (NGA), and Uzbekistan (UZB) experiencing a high impact, while others like Georgia (GEO) and Mauritius (MUS) seem to have been less affected.

The capacity of countries to respond is evident in the differences between high-income and low-income countries. High-income countries generally have more resources to allocate to social safety net programs, while low-income countries struggle to provide substantial support.

In conclusion, the data reveals a complex picture of how different countries responded to the economic challenges of the COVID-19 crisis. The effectiveness of social safety nets seems to be closely tied to a country’s income level and its ability to allocate resources to support its citizens. However, there are notable exceptions and variations within income groups, highlighting the importance of specific government policies and the unique circumstances of each country.

By examining the prevalence of income reduction and the role of social assistance, this chapter aims to provide insights into the effectiveness of social protection measures in addressing the economic fallout of the pandemic and to identify areas for improvement in future crisis response.

## 7. Comparative analysis of high-income, middle-income, and low-income countries

This chapter delves into a comparative analysis of how high-income, upper middle-income, lower middle-income, and low-income countries have fared in terms of income insecurity and social protection responses during the COVID-19 pandemic.

**Fig 4:**
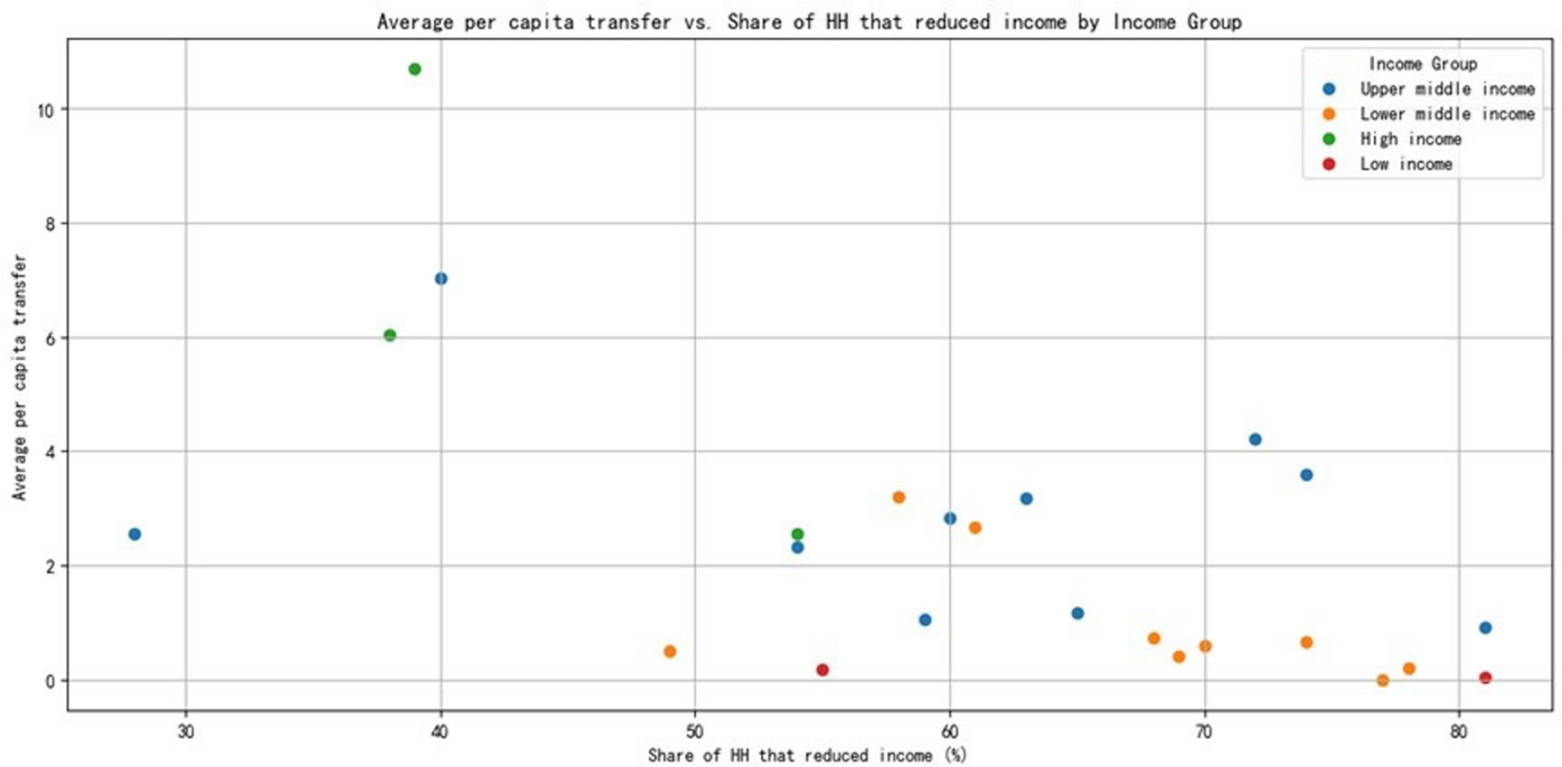
Average per capita transfer VS share of HH that reduced income.

- High-income countries generally have higher per capita transfers: Countries like Chile (CHL) and Mauritius (MUS) have higher average per capita transfers compared to middle-income and low-income countries. This is likely due to the higher cost of living and more robust social protection programs in these countries.
- Household income reduction is significant across all income groups: countries such as Honduras (HND), Nigeria (NGA), and Malawi (MWI) report high percentages of households with reduced income, indicating the widespread impact of the COVID-19 crisis on the economic well-being of people across different income levels.
- Lower-middle-income countries show variability in social protection response: While some countries like Uzbekistan (UZB) have a relatively high average per capita transfer, others like Ghana (GHA) have very low figures, indicating differences in the capacity and effectiveness of social safety nets in Fig 4.

### 7.1. High-income countries: Robust safety nets and challenges

High-income countries typically have more robust social safety nets due to their greater financial resources and more developed welfare systems. This section explores the extent to which these safety nets have buffered the impact of the pandemic on income insecurity. It also examines the challenges faced by high-income countries, such as the scale of financial support needed, the digital divide in accessing benefits, and the sustainability of increased social spending.

**Income Reduction Rate of Households**: During the COVID-19 pandemic, many countries experienced household income losses. Low-income countries had a higher proportion of households experiencing income reduction, with Malawi (MWI) and Nigeria (NGA) reporting 81% and 78% respectively. Upper-middle-income countries like Argentina (ARG) and Georgia (GEO) had relatively lower rates, standing at 40% and 28% respectively.

**Strength of Social Safety Nets**: The average per capita transfer payments indicate that high-income countries have relatively stronger social safety nets. For instance, Poland (POL) has an average per capita transfer payment of 10.68907329, while Mauritius (MUS) stands at 6.043621576. In contrast, this figure is generally lower for low-income and lower-middle-income countries, and some even record zero.

**Impact of the COVID-19 Crisis**: Due to their weak economic foundations and insufficient social safety nets, low-income countries have a poor ability to resist the pandemic, resulting in a higher proportion of households experiencing income reduction. Although upper-middle-income countries also saw a decrease in household income, their relatively sound social safety nets and transfer payments have mitigated the impact of the pandemic on households to a certain extent.

1. **Changes in Average Per Capita Transfer Amounts over Time across Different Income Groups**:
  - High-income countries’ average per capita transfer amounts in 2015 and 2017 were 10.69 and 4.30 respectively.
  - Low-income countries’ average per capita transfer amounts in 2016 and 2018 were 0.04 and 0.17 respectively.
  - Lower-middle-income countries’ average per capita transfer amounts in 2015, 2016, 2017, and 2018 were 1.86, 0.33, 0.74, and 0.97 respectively.
  - Upper-middle-income countries’ average per capita transfer amounts in 2016 and 2017 were 2.55 and 0.92 respectively.
2. **Correlation between Average Per Capita Transfer Amounts and the Proportion of Households with Reduced Income in Each Country**: The correlation coefficient is -0.60, indicating a moderate negative correlation between these two variables. This means that, generally speaking, the higher the proportion of households experiencing income reduction, the lower the average per capita transfer amount.

### 7.2. Upper middle-income countries: Balancing growth and security

Upper middle-income countries often face the dual challenge of maintaining economic growth while providing adequate social protection. This section analyzes how these countries have balanced these priorities during the pandemic. It looks at the effectiveness of their social protection programs, the role of fiscal policy in supporting both growth and security, and the innovative approaches adopted to address income insecurity amidst economic constraints.

### 7.3. Lower middle-income and low-income countries: Vulnerabilities and adaptations

Lower middle-income and low-income countries are particularly vulnerable to economic shocks due to their weaker health systems, limited social protection infrastructure, and higher reliance on informal labor markets. This section assesses how these countries have adapted to the pandemic, highlighting the strategies used to mitigate income insecurity with limited resources. It also examines the role of international aid and cooperation in supporting social protection efforts in these countries.

Throughout this comparative analysis, the chapter aims to identify commonalities and differences in the experiences of countries across income groups. It seeks to understand the factors that have influenced the outcomes for income insecurity and social protection, and to draw lessons that can inform more effective and equitable crisis response strategies in the future.

## 8. Discussion

**Fig 5:**
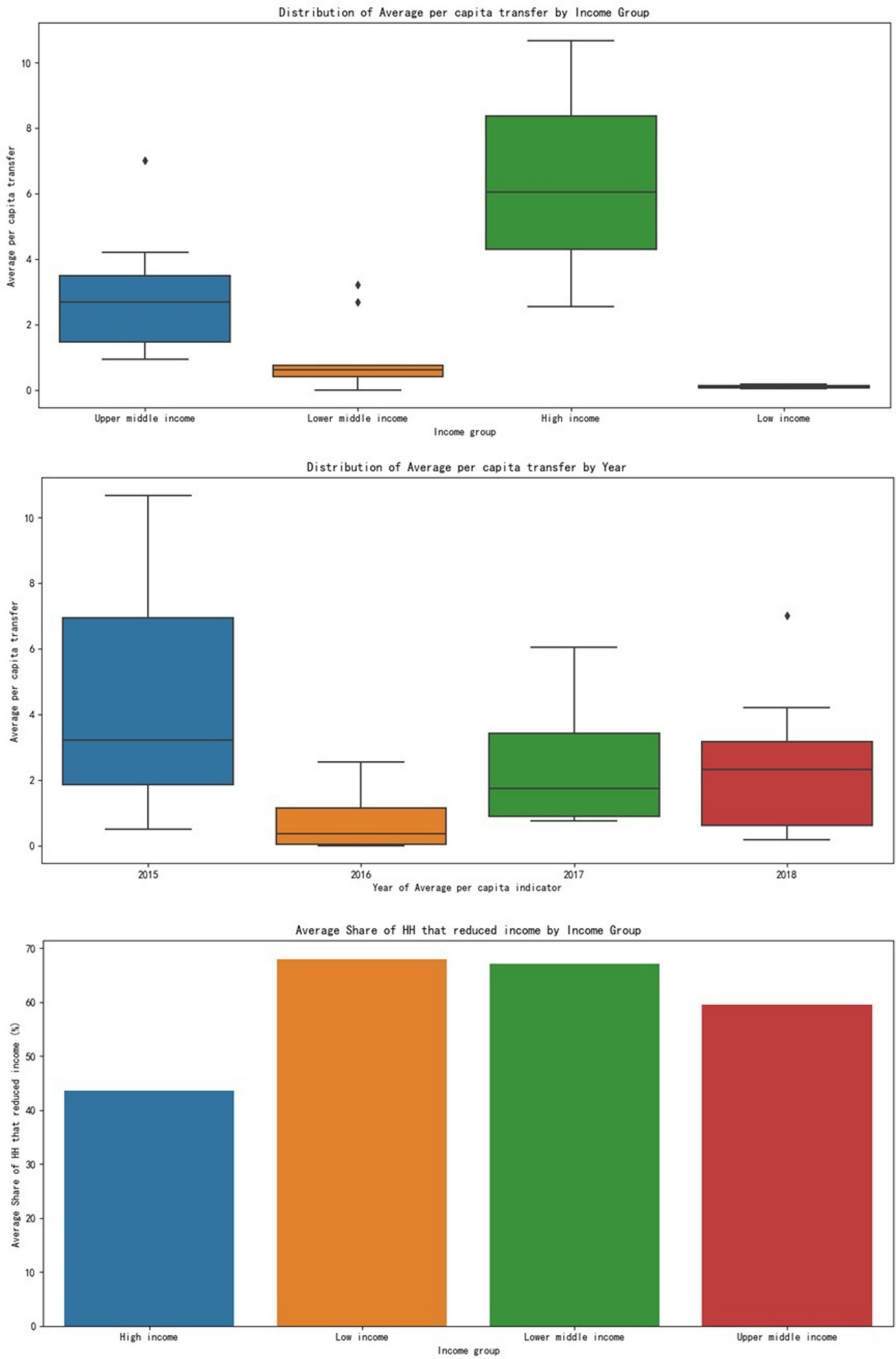
Changes in average income reduction rate of households over time across different income groups.

This section of the paper provides a critical analysis of the study’s findings, acknowledges the strengths and limitations of the research, and offers policy recommendations based on the research findings in Fig 5.

1. **Changes in Average Income Reduction Rate of Households over Time across Different Income Groups**:
  - High-income countries’ average income reduction rate of households in 2015 and 2017 was 39% and 46% respectively.
  - Low-income countries’ average income reduction rate of households in 2016 and 2018 was 81% and 55% respectively.
  - Lower-middle-income countries’ average income reduction rate of households in 2015, 2016, 2017, and 2018 was 53.5%, 75.5%, 68.0%, and 69.5% respectively.
  - Upper-middle-income countries’ average income reduction rate of households in 2016 and 2017 was 28% and 81% respectively.
2. **Correlation between Average Per Capita Transfer Amounts and the Proportion of Households with Reduced Income in Each Country**: The correlation coefficient is -0.60, indicating a moderate negative correlation between these two variables. This means that, generally, the higher the proportion of households experiencing income reduction, the lower the average per capita transfer amount.
3. **Box Plot Showing the Distribution of Average Per Capita Transfer Amounts across Different Income Groups**: From the box plot, we can observe the distribution of average per capita transfer amounts across different income groups, including the median, quartiles, and potential outliers.
4. **Distribution of Average Per Capita Transfer Amounts across Different Years**: Through the box plot, we can visualize the distribution of average per capita transfer amounts in different years, encompassing the median, quartiles, and potential outliers.
5. **Average Income Reduction Rate of Households across Different Income Groups**:
  - The average income reduction rate of households in high-income countries is 43.67%.
  - The average income reduction rate of households in low-income countries is 68.00%.
  - The average income reduction rate of households in lower-middle-income countries is 67.11%.
  - The average income reduction rate of households in upper-middle-income countries is 59.60%.

### 8.1. Key findings and their implications

1. The discussion begins by presenting the key findings of the study, which include the prevalence of income insecurity across income groups, the effectiveness of social protection measures in mitigating income loss, and the variations in social protection responses across high-income, middle-income, and low-income countries. The implications of these findings are then explored, with a focus on how they inform our understanding of the impact of the COVID-19 pandemic on income insecurity and the role of social protection in crisis response. Income Groups categorizes countries into four income groups ‐ High income, Upper middle income, Lower middle income, and Low income. This classification is likely based on the World Bank’s income group classification, which is typically updated annually.
2. Share of HH that reduced income: This column indicates the percentage of households in each country that experienced a reduction in income due to the COVID-19 crisis. For example, in ARM (Armenia), 54% of households saw their income reduced.
3. Average per capita transfer: The last column shows the average amount of money (presumably in US dollars) transferred to individuals as part of social protection measures. For instance, in ARG (Argentina), the average per capita transfer was $7.026 in 2018.
4. Year of Average per capita indicator: This indicates the year for which the average per capita transfer data is reported, which varies by country.

The findings show that in countries with limited social safety nets and a large share of employment in the informal sector, household incomes were particularly hard-hit. This aligns with the social investment perspective, which argues that the lack of social safety nets and employment protection reduces the ability of individuals to recover from shocks.

### 8.2. Strengths and limitations of the study

The interpretation of the results suggests that the government response to the pandemic, particularly the phasing out of income support programs before household earnings fully recovered, increased the vulnerability of households. This is consistent with the social insurance perspective, which emphasizes the importance of collective risk management through social insurance programs.

The discussion then addresses the strengths and limitations of the study. The strengths may include the use of a comprehensive dataset, the application of mixed-methods research design, and the cross-country comparative analysis. The limitations could include the availability of data, the selection of countries, and the potential for confounding factors. Acknowledging these limitations is crucial for contextualizing the findings and understanding their limitations in real-world applications.

### 8.3. Policy recommendations for enhanced social protection

Based on the study’s findings, the discussion concludes with policy recommendations for enhancing social protection in response to future crises. These recommendations may include:

- Strengthening social safety nets to ensure greater coverage and effectiveness in times of crisis.
- Investing in digital infrastructure to improve access to social protection benefits, particularly in rural and remote areas.
- Targeting social protection programs to vulnerable groups, such as informal workers and those in low-income sectors.
- Promoting international cooperation and coordination to support social protection efforts in lower-income countries.
- Conducting regular assessments of social protection systems to identify gaps and adapt to changing economic and social needs.

The policy recommendations aim to provide actionable insights for policymakers and stakeholders to improve the resilience of social protection systems and reduce income insecurity during future crises.

The discussion section of the paper explores the implications of the findings for social protection policies and the need for more comprehensive social safety nets to reduce household vulnerability and financial risk.

In conclusion, the research demonstrates the importance of integrating social protection theories into the analysis of government responses to income shocks. By doing so, we can gain a deeper understanding of the impact on households and the potential spillover effects on financial institutions. This understanding can inform the design of more effective social protection policies to mitigate the impact of future crises.

## 9. Conclusion

The conclusion of the paper summarizes the study’s main contributions, outlines potential future research directions, and offers final thoughts on the broader implications of income insecurity and social protection in the context of the COVID-19 pandemic.

### 9.1. Summary of the study’s main contributions

This section succinctly recaps the key findings and insights of the study. It highlights the study’s contributions to the understanding of income insecurity during the pandemic, the role of social protection, and the variations in responses across income groups. The conclusion emphasizes the importance of these findings for policymakers, practitioners, and researchers in the field of social protection and crisis response.

The average per capita transfer values from social protection and labor programs for various countries during a specific year, often during or preceding the COVID-19 crisis. The data is segmented by income group (High, Upper-middle, Lower-middle, and Low) and includes the share of households that reduced their income, likely due to economic impacts from the pandemic.

1. **Varying levels of support**: The average per capita transfer values vary widely across countries, indicating differing levels of social protection programs and resources. High-income countries tend to have higher transfer values, while low-income countries have significantly lower values.
2. **Impact of income group**: As expected, the average per capita transfer tends to be higher in higher-income countries and lower in lower-income countries. This reflects the different levels of fiscal capacity and resources available for social protection programs.
3. **High shares of income reduction**: The share of households that reduced their income is high across all income groups, indicating the widespread economic impact of the COVID-19 crisis. This underscores the importance of robust social protection programs to mitigate the effects of such crises.
4. **Need for enhanced social protection**: The low transfer values in low- and lower-middle-income countries suggest that these countries may need to enhance their social protection systems to better support vulnerable populations during economic crises.
5. **Importance of timely and targeted support**: The data highlights the need for timely and targeted social assistance to reach households experiencing income losses. Swift and effective action by governments can help mitigate the negative impacts of economic shocks.
6. **Room for policy improvements**: While the data shows the existence of social safety nets, the differing levels of support indicate room for policy improvements to ensure equitable and adequate social protection for all.

In summary, the data highlights the importance of robust social protection programs, particularly during economic crises such as the COVID-19 pandemic. Governments should prioritize enhancing their social safety nets to better support vulnerable populations and mitigate the negative impacts of such crises. These findings have important implications for policy-makers, highlighting the need for robust and adaptable social protection systems that can effectively respond to economic shocks like the COVID-19 pandemic. They also underscore the importance of targeted interventions to address within-country inequalities and the differential impacts on various demographic groups.

### 9.2. Future research directions

The conclusion also suggests areas for future research that build upon the study’s findings. These directions may include:

- Longitudinal studies to track the evolution of income insecurity and social protection responses over time.
- Explorations of the psychological and social impacts of income insecurity during and after the pandemic.
- Comparative analyses of the effectiveness of different social protection programs and policies.
- Studies that consider the intersection of income insecurity with other dimensions of inequality, such as gender, race, and ethnicity.

### 9.3. Final thoughts on income insecurity and social protection

In the final section, the conclusion offers a broader reflection on the significance of income insecurity and social protection during the COVID-19 pandemic. It emphasizes the critical role of social protection in providing a safety net for individuals and households during times of crisis and the importance of addressing income insecurity to promote social cohesion and economic stability. The conclusion underscores the need for ongoing research and policy action to strengthen social protection systems and ensure the resilience of communities in the face of future challenges.
